# Positive surgical margin’s impact on short-term oncological prognosis after robot-assisted partial nephrectomy (MARGINS study: UroCCR no 96)

**DOI:** 10.1038/s41598-022-23146-4

**Published:** 2022-10-31

**Authors:** Arnoult Morrone, Imad Bentellis, Jean-Christophe Bernhard, Karim Bensalah, Cécile Champy, Franck Bruyere, Nicolas Doumerc, Jonathan Olivier, François Audenet, Bastien Parier, Martin Brenier, Jean-Alexandre Long, François-Xavier Nouhaud, Nicolas Branger, Hervé Lang, Thomas Charles, Evanguelos Xylinas, Thibaut Waeckel, Florie Gomez, Romain Boissier, Benjamin Rouget, Aysha Shaikh, Daniel Chevallier, Damien Ambrosetti, Matthieu Durand

**Affiliations:** 1 Urology, Andrology, Renal Transplant Unit, Hôpital Pasteur 2, Nice University Hospital, Nice, France; 2 Department of Urology, Bordeaux University Hospital, Bordeaux, France; 3 Department of Urology, Rennes University Hospital, Rennes, France; 4 Department of Urology, Henri Mondor University Hospital, APHP, Paris, France; 5 Department of Urology, Tours University and Regional Hospital, Tours, France; 6 Department of Urology and Renal Transplantation, Toulouse University Hospital, Toulouse, France; 7 Department of Urology, Lille University and Regional Hospital, Lille, France; 8 Department of Urology, AP-HP Centre, Hôpital Européen Georges Pompidou, Université de Paris, Paris, France; 9 Department of Urology, Hôpital Bicêtre, Université Paris Saclay, APHP, Le Kremlin-Bicêtre, France; 10 Department of Urology, Paris Saint-Joseph Hospital Group, Paris, France; 11 Department of Urology, Grenoble University Hospital, Grenoble, France; 12 Department of Urology, Rouen University Hospital, Rouen, France; 13 Department of Urology, Institut Paoli-Calmettes, Marseille, France; 14 Department of Urology, Strasbourg University and Regional Hospital, Strasbourg, France; 15 Department of Urology, Poitiers University Hospital, Poitiers, France; 16 Department of Urology, Bichat-Claude Bernard Hospital, APHP, Paris Descartes University, Paris, France; 17 Department of Urology, Caen University Hospital, Caen, France; 18 Department of Urology, Tenon Hospital, APHP, Paris, France; 19 Department of Urology and Renal transplantation, La Conception University Hospital, Aix-Marseille University, APHM, Marseille, France; 20 Department of Urology, Libourne Hospital, Libourne, France; 21 Central Laboratory of Pathology, Nice University Hospital, Nice, France; 22 INSERM U1081 - CNRS UMR 7284, Nice University Côte d’Azur, Nice, France; 23 Urology, Andrology, Renal Transplant Unit, Hôpital Pasteur 2, Nice University Hospital, 30 voie Romaine, 06000 Nice, France

**Keywords:** Renal cell carcinoma, Surgical oncology, Risk factors

## Abstract

The oncological impact of positive surgical margins (PSM) after robot-assisted partial nephrectomy (RAPN) is still under debate. We compared PSM and Negative Surgical Margins (NSM) in terms of recurrence-free survival (RFS), metastasis-free survival (MFS) and overall survival (OS) after RAPN, and we identified predictive factors of PSM. Multi-institutional study using the UroCCR database, which prospectively included 2166 RAPN between April 2010 and February 2021 (CNIL DR 2013-206; NCT03293563). Two groups were retrospectively compared: PSM versus NSM. Prognostic factors were assessed using Kaplan–Meyer curves with log-Rank test, cox hazard proportional risk model and logistic regression after univariate comparison. 136 patients had PSM (6.3%) and 2030 (93.7%) had NSM. During a median follow-up of 19 (9–36) months after RAPN, 160 (7.4%) recurrences were reported. Kaplan–Meier curves and analysis suggested that RFS, MFS and OS were not affected by a PSM (*p* = 0.68; 0.71; 0.88, respectively). In multivariate analysis predictors of PSM were a lower RENAL score (*p* = 0.001), longer warm ischemia time (WIT) (*p* = 0.003) and Chromophobe Renal Cell Carcinoma (chrRCC) (*p* = 0.043). This study found no impact of PSM on RFS, MFS or OS, and predictors of PSM were the RENAL score, WIT and chrRCC.

## Introduction

Surgically resected tumors show a lower TNM stage than was the case 20 years ago, thanks to routine cross-section imaging^1,2^. Partial nephrectomy (PN), whenever possible, is the reference treatment since 2010 to date^3,4^. The proliferation of robotic surgical platforms has resulted in an exponential increase in the number of patients undergoing robotic‐assisted partial nephrectomy (RAPN)^5^.

The aim of this nephron-sparing surgery (NSS) is an optimal oncological control while preserving overall renal function, without any post-operative complication. However, its main pitfall remains the possibility of positive surgical margins, occurring in 0.1–10.7% of cases^6^.

European guidelines recommend intensified follow-up after PSM encounter but there is no consensus for a particular strategy and the association between PSM and recurrence is still under debate^3,7^. The majority of studies reported so far have indicated that positive surgical margins do not correlate with a higher risk of metastases or decreased cancer specific survival (CSS)^8,9^. On the other hand, large retrospective studies showed that PSM, are an independent predictor of recurrence^10,11^. Absence of PSM, such as in *Trifecta*^12^ achievement, has also shown a predictive role on long term outcomes after RAPN^13^.

The objective of this study was to compare the oncological outcomes of patients undergoing RAPN for RCC according to the surgical margin status, and to identify factors associated with PSM.

## Patients and methods

### Study design

After institutional review board approval, the MARGINS study was conducted in the framework of the UroCCR project (NCT03293563). All patients were given oral and written information about the objectives and methodology of the UroCCR project, and data of those who provided written and informed consent were prospectively included in the UroCCR database. All experimental protocols were approved by the ethics committee of Commission Nationale de l'Informatique et des Libertés (CNIL authorization number DR-2013-206). We reviewed the medical records of all patients in this database, between April 2010 and February 2021 in 20 French centers.

Given the retrospective multicenter study design, the surgical techniques and preoperative work-up were not standardized across centers, but European Guidelines^3^ were followed. The following preoperative data were collected for each patient: age at RAPN, sex, BMI, ECOG and ASA score, Tumor characteristics, including the clinical TNM stage, clinical tumor size, tumor side and radiological RENAL nephrometry score. All patients had pre-operative CT scan including abdominal and pelvic sequences for evaluation of the tumor, most of the time with thoracic sequences for assessment of extension.

The following surgical data were recorded: indication of NSS (elective, imperative, relative), surgical approach (transperitoneal (TP), retroperitoneal (RP)), overall operative time, type of clamping, warm ischemia time (WIT), estimated blood loss, peri-operative complications and conversion to open surgery. The surgical approach was considered discordant when the tumor was anterior with an RP approach and inversely for posterior tumors with a TP approach.

Pathology characteristics included histologic subtype, pTNM, diameter and grade and UISS prognostic category. Post-operative complications were recorded according to current guidelines^14^ and graded according to the Clavien–Dindo classification^15^.

Follow-up protocols were similar across centers. It involved a clinical interview, a physical examination, serum creatinine, a CT scan to assess local recurrence and metastasis progression. Median follow-up was calculated as the median time between surgery and the last consultation.

### Outcomes of interest

The primary endpoint was PSM, which was defined as the presence of cancer cells at the margin of the surgical specimen reported for H&E stained tissues.

### Statistical analysis

Means and standard deviations were reported for continuous variables, and proportions for nominal variables. The exact Fisher test was used to compare nominal variables, the Kruskal–Wallis test for ordinal variables and the Student test to compare quantitative continuous variables. Non-parametric tests were used for small sample sizes. The probabilities of OS, RFS and MFS were estimated using the Kaplan–Meier method. Multivariable Cox regression analyses were used to seek predictors of OS, RFS and MFS. Only variables with a *p*-value < 0.25 in univariate analysis were included in the multivariate model. Collinearities were included in the initial model, and the final model was built with factors restricted to those reported in the existing literature (larger size or RENAL score, pT2, grade III-IV, overall operative time, blood loss). R statistical software was used for the statistical analyses^16^. All tests were two-sided with a significance level at *p* < 0.05. All methods were performed in accordance with the relevant guidelines and regulations.

## Research involving human participants, their data or biological material and informed consent

All patients were given oral and written information about the objectives and methodology of the UroCCR project, and data of those who provided written consent were prospectively included in the UroCCR database (CNIL authorization number DR-2013-206; NCT03293563). The datasets used and analyzed during the current study are available from the corresponding author on reasonable request.

## Results

### Patients’ characteristics

**Figure 1:**
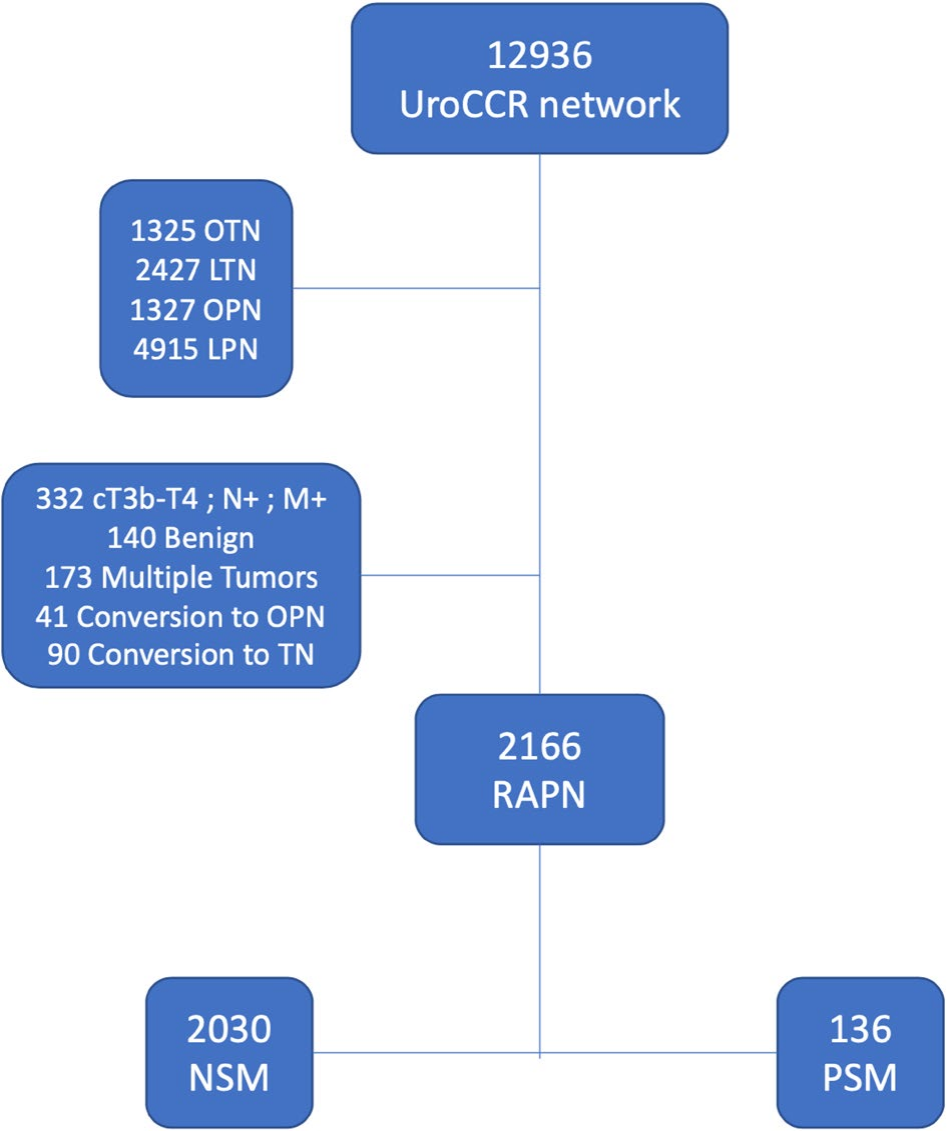
Flowchart. *OPN* open partial nephrectomy, *LPN* laparoscopic partial nephrectomy, *OTN* open total nephrectomy, *LTN* laparoscopic total nephrectomy, *TN* total nephrectomy, *RAPN* robot-assisted partial nephrectomy, cTNM M + , metastasis at diagnosis, *PSM* Positive surgical margin, *NSM* negative surgical margin.

We reviewed the medical records of the 12,936 patients of the UroCCR network, including hereditary syndromes if known at baseline, and excluding patients with previous history of RCC. First, we excluded those who underwent open partial or radical nephrectomy (OPN n = 1327, ORN n = 1345), then those who had laparoscopic partial or radical nephrectomy (LPN n = 4915, LRN n = 2427). Of the 2922 patients who underwent RAPN over the study period, 2166 met the inclusion criteria. Exclusion criteria were cT3b-4, cN + or cM + (n = 332), conversion to OPN (n = 41), conversion to radical nephrectomy (n = 90), benign (n = 140) or multiple (n = 173) tumors. (Fig. 1).

Of the 2166 patients included, 136 patients had PSM (6.3%) and 2030 (93.7%) had NSM. Patients’ characteristics are summarized in Table 1. The mean age at surgery (PSM: 60.2 vs. NSM: 59.9, *p* = 0.094), ASA (*p* = 0.4), cTNM (*p* = 0.987) were similar in both groups. The RENAL Score was lower in the PSM group (PSM: 6 vs. NSM: 7, *p* = 0.05). The overall median follow-up was 19^9,36^ months; 160 (7.4%) recurrences (88 local and 72 metastases) were reported: 9 (6.6%) in the PSM group and 151 (7.4%) in the NSM group.

**Table 1:** Patients Characteristics at time of RAPN.

	NSM	PSM	*p*
n	2030	136	
Sex male, n (%)	608 (30.0)	31 (22.8)	0.094
Age, year (SD)	59.90 (12.41)	60.17 (11.59)	0.805
BMI, kg/m^2^ (SD)	27.57 (5.45)	27.40 (5.67)	0.727
ASA, n (%)			0.400
1	495 (26.0)	25 (19.5)	
2	1062 (55.8)	76 (59.4)	
3	336 (17.7)	26 (20.3)	
4	9 (0.5)	1 (0.8)	
ECOG, n (%)			0.032
0	1366 (80.4)	76 (69.7)	
1	271 (15.9)	27 (24.8)	
2	53 (3.1)	4 (3.7)	
3	10 (0.6)	2 (1.8)	
cTNM: T, n (%)			0.987
T1a	1312 (67.2)	87 (67.4)	
T1b	627 (32.1)	41 (31.8)	
T2a	13 (0.7)	1 (0.8)	
cTNM : Nx (%)	116 (5.7)	9 (6.6)	0.805
cTNM : Mx (%)	215 (10.6)	15 (11.0)	0.987
Tumor size, cm (SD)	3.51 (1.39)	3.60 (1.40)	0.514
Median RENAL Score	7	6	0.05

*NSM* negative surgical margins, *PSM* positive surgical margins, *BMI* body mass index.

### Perioperative outcomes

PSM was associated with a longer mean WIT (PSM: 20.4 ± 10.3 min vs. NSM: 17.8 ± 8.5 min, *p* = 0.001). Complications during surgery (PSM: 6.7% vs. NSM: 3.9%, *p* = 0.168), retroperitoneal approach (n = 168) (PSM: 10.9% vs. NSM: 7.8%, *p* = 0.279), discordant approaches (PSM: 41.2% vs. NSM: 42.8%, *p* = 0.845) and on-clamp procedure (PSM: 91% vs. NSM: 86.8%, *p* = 0.198) were not associated with PSM. Intraoperative data are shown in Table 2.

**Table 2:** Perioperative outcomes.

	NSM	PSM	*p*
n	2030	136	
Retroperitoneal approach, n (%)	154 (7.8)	14 (10.9)	0.279
Discording approach, n (%)	686 (42.8)	40 (41.2)	0.845
NSS (%)			0.568
Elective	1389 (78.2)	102 (82.3)	
Impérative	144 (8.1)	8 (6.5)	
Relative	243 (13.7)	14 (11.3)	
On-clamp (%)	1741 (86.8)	122 (91.0)	0.198
WIT, min (SD)	17.76 (8.45)	20.43 (10.30)	0.001
Operative time, min (SD)	161.93 (89.28)	161.25 (58.50)	0.935
Blood loss, mL (SD)	258.15 (313.17)	281.67 (415.07)	0.424
Complication during surgery, n (%)	78 (3.9)	9 (6.7)	0.168
Conversion to OPN, n (%)	23 (1.1)	0 (0.0)	0.415

*NSM* negative surgical margins, *PSM* positive surgical margins, *NSS* nephron sparing surgery, *WIT* warm ischemia time, *OPN* open partial nephrectomy.

### Pathology findings

chrRCC (PSM: 19.9% vs. NSM: 8.5%, *p* < 0.001) was significantly associated with PSM, whereas the ccRCC histology type (PSM: 56.6% vs. NSM: 70.3%, *p* = 0.001) was associated with a lower likelihood of PSM.

Results are shown in Table 3.

**Table 3:** Histological outcomes.

	NSM	PSM	*p*
n	2030	136	
ccRCC n (%)	1427 (70.3)	77 (56.6)	0.001
chrRCC n (%)	172 (8.5)	27 (19.9)	< 0.001
papRCC n(%)	333 (16.4)	27 (19.9)	0.354
Tumor size (cm)	3.29 (1.44)	3.44 (1.43)	0.220
Fuhrman (%)			0.001
1	115 (5.8)	16 (12.5)	
2	1035 (52.6)	60 (46.9)	
3	529 (26.9)	31 (24.2)	
4	289 (14.6)	21 (16.4)	
UISS prognosis risk, n (%)			0.114
Low	935 (47.9)	52 (40.3)	
Moderate	1017 (52.1)	77 (59,7)	
Leibovich risk, n (%)			0.508
Low	1523 (75.0)	102 (75.0)	
Intermediate	152 (7.5)	7 (5.1)	
High	355 (17.5)	27 (19.9)	

*NSM* negative surgical margins, *PSM* positive surgical margins, *ccRCC* clearcell renal cell carcinoma, *chRCC* chromophobe renal cell carcinoma, *papRCC* papillary renal cell carcinoma.

**Figure 2:**
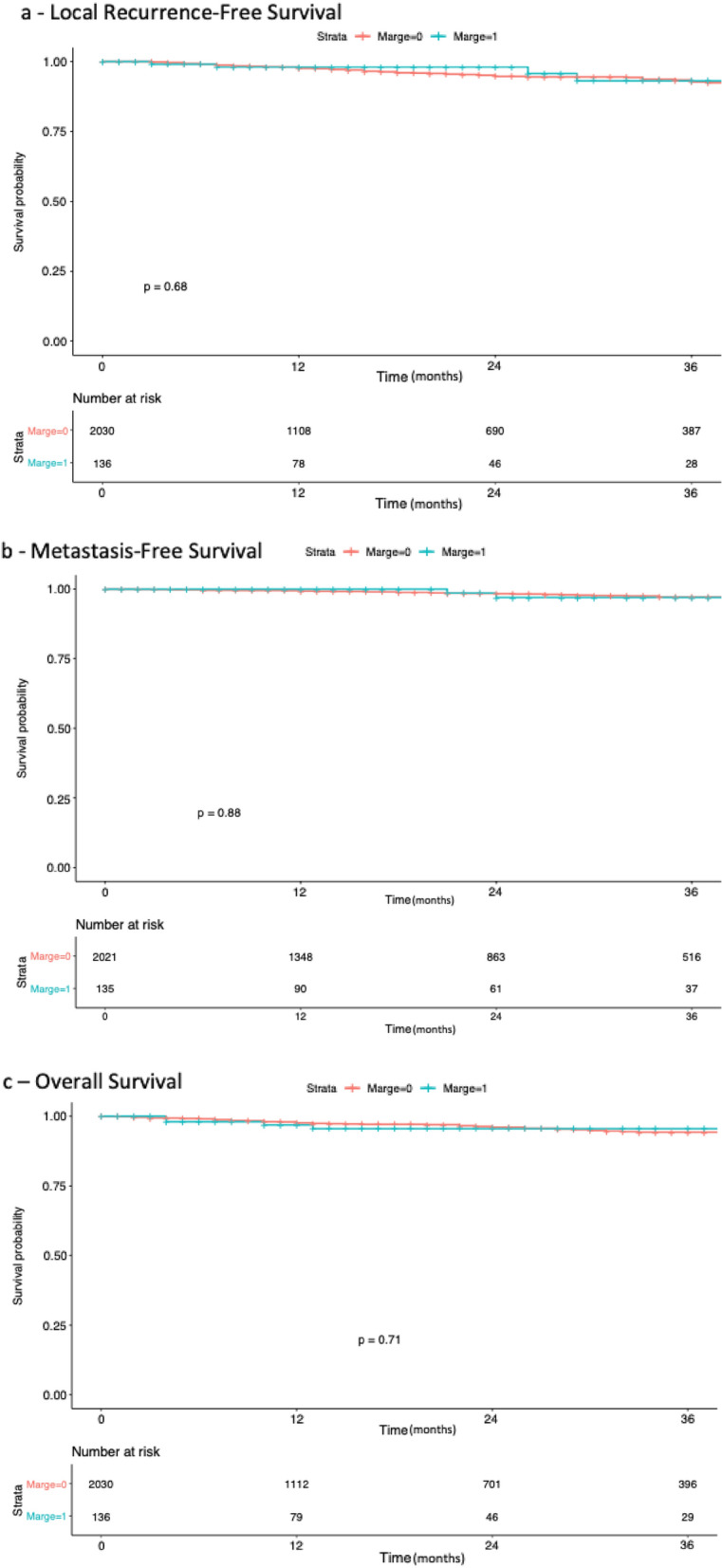
Kaplan–Meier survival curves after RAPN according to surgical margin. (**a**) Local Recurrence-Free Survival, (**b**) Metastasis-Free Survival, (**c**) Overall Survival. No statistical difference was made between groups.

The median follow-up was 21.5 mo [9–37] for PSM: versus 19 mo [9–36] for NSM (*p* = 0.662). Survival analysis did not show any difference between the groups for RFS (Fig. 2a), MFS or OS (Fig. 2b,c) (respectively *p* = 0.68; 0.71; 0.88).

**Figure 3:**
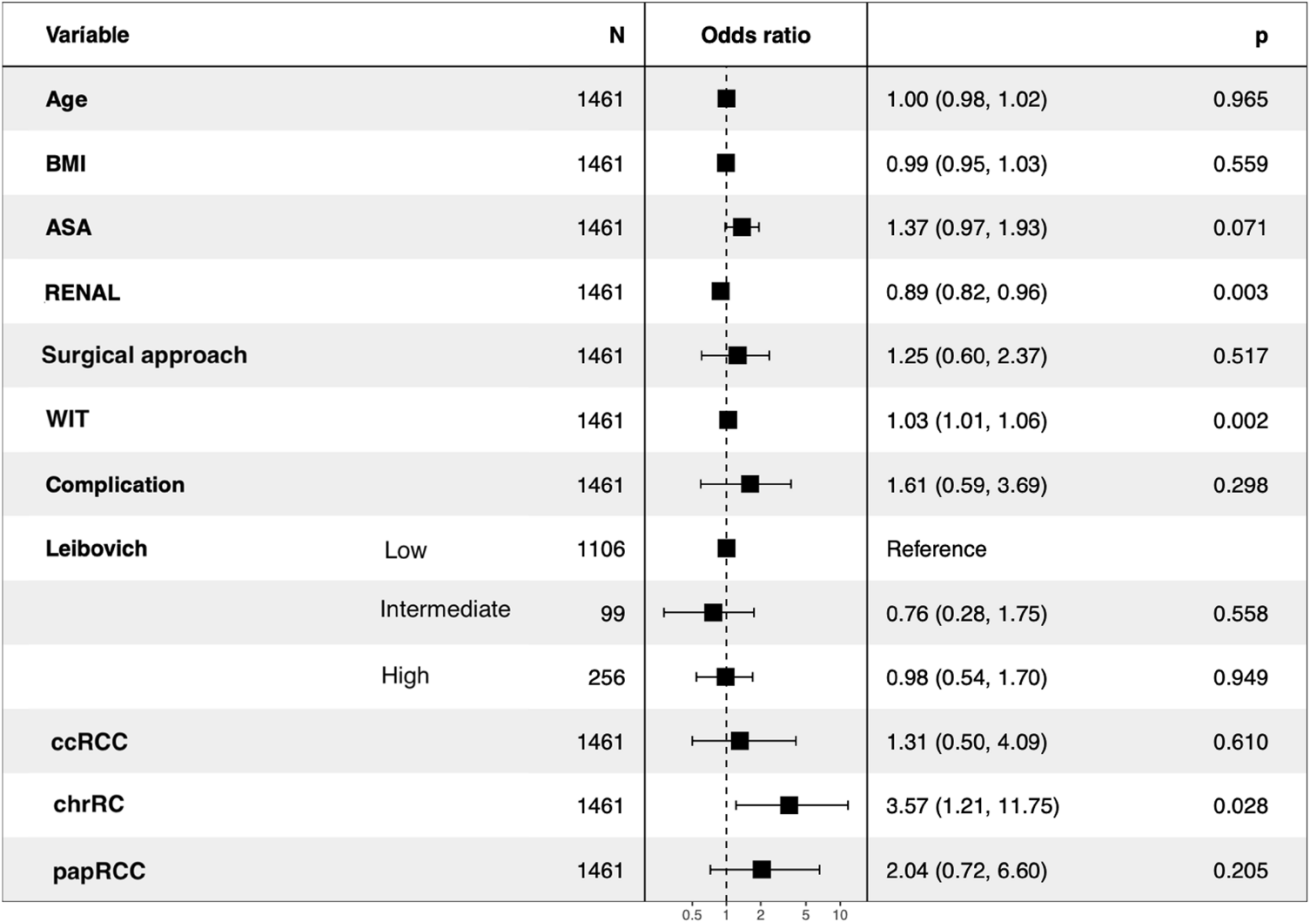
Multivariable analysis of risk factors for positive margin. Lower RENAL Score, Longer WIT and chrRCC are prognostic factors of PSM.

Multivariate analysis (Fig. 3) showed that a lower RENAL score (*p* = 0.001), a longer warm ischemia time (WIT) (*p* = 0.003) and chrRCC histology (*p* = 0.043) were predictors of PSM.

## Discussion

Early-stage diagnosis in RCC and the diagnosis of small renal masses have led to an evolution in surgical approaches. Since 2010 to date, for cT1-T2 renal mass, PN and then RAPN rather than OPN, when feasible, is the recommended surgical approach^3,4,17^. Therefore, it had no impact on clinical practices over the years for the authors. In order to achieve its nephron-sparing potential, RAPN has to meet three main challenges: WIT < 25 min to prevent renal failure, oncological control, currently evaluated as NSM, and no perioperative complications.

Although a PSM could be regarded as cancer cells remaining in the kidney, hemostasis may induce ischemia and necrosis in these cells. In addition, tumor cells in the surgical specimen may be in tangential contact with the margin, corresponding to a PSM, but with no cancer cells remaining in the resection bed. As for NSM, the analysis of frozen sections during tumor resection led to 5% of false-negative NSM^18^, and an NSM is no guarantee that a recurrence will not occur^7^. Given the above, the prognostic value of PSM after RAPN is still a matter of debate.

We found 6.3% of PSM, which is in line with the 0.1–10.7% reported in the literature^6^, but local recurrence occurred in only 5.9% (N = 8) of our PSM patients. Our median follow-up was 19 months, which is equivalent to the median time to recurrence after PN (19 mo) according to Takagi et al.^19^. In the literature, there is no consensus on the presence or absence of a statistical correlation between surgical margins and recurrence rates or specific survival. On one hand, Petros et al*.* showed a close association between PSM and disease progression after PN^20^ in a large single-center cohort of 2297 PN with 1863 (81%) RCC and 34 (1.48%) PSM. The 34 PSM patients were matched 1:3 with 100 NSM patients for tumor size, RENAL score, grade, and pathologic stage. They found that PSM had an impact on 5-year survival probability for OS (0.99 for NSM vs. 0.97 for PSM), local RFS (0.98 vs. 0.77), and MFS (0.95 vs. 0.84). Moreover, in another analysis, Wood et al*.*^21^ demonstrated a strong association between PSM at the time of PN and local tumor bed recurrence after a median time of 23mo: 15.9% of PSM in the recurrence group versus 3% in the control group. Khalifeh et al*.*^22^ evaluated the oncological outcomes of 21 PSM among 947 RAPN for malignant tumors with 13 months of median follow-up. Following the 947 procedures, there were nine recurrences and four cases of metastases. PSM were strongly linked to recurrence, with an HR of 18.4 after adjustment. The 3-year recurrence-free rate and metastasis-free survival rate were lower in the PSM group than in the NSM group (respectively 47% vs. 98.3% and 63% vs. 99.5%). In a retrospective study of 1240 PN for RCC with 97 PSM leading to 67 recurrences, Shah et al*.*^23^ demonstrated that PSM was an independent risk factor for recurrence. In their subgroup analysis, PSM was found to be a risk factor for recurrence in high-risk tumors (pT2-3a or grades III–IV) but not in low-risk tumors (pT1 or grades I–II). Moreover, Bernhard et al*.*^24^ evaluated the predictive factors for ipsilateral recurrence in 809 NSS procedures with 27 months of follow-up. They reported 26 local recurrences and 15.4% of PSM. Multivariate analysis showed a link between PSM and local recurrence with an HR of 11.5 (*p* < 0.01).

In contrast, Bensallah et al*.*^8^ conducted a matched-pair study that included 101 PSM and 102 NSM matched for surgical indication, tumor size, and tumor grade. PSM had no influence on 5-year RFS, 5-year cancer specific survival (CSS) or 5-year OS. Yossepowitch et al*.*^25^ studied 77 PSM in 1390 PN with a median follow-up of 40.8 months and found that PSM were not associated with worse RFS or MFS. In addition, Rothberg et al*.*^26^ compared 797 NSM with 42 PSM after RAPN and reported that oncological outcomes in PSM patients were no worse than those in NSM patients after a median follow-up of 18.8 months. The outcomes in our study are in line with those of the Rothberg study in that we found no statistical association between PSM and RFS, MFS or OS.

Multivariable analysis showed that higher RENAL scores were associated with NSM. This paradoxical finding could be because smaller masses within the renal parenchyma may be harder to find, and the surgeon might feel overconfident during such a PN procedure, which in theory should be technically easier given the small size of the tumor. This hypothesis is supported by Schiavana et al.^27^, who founded that smaller tumors cT1a versus cT2, laparoscopic technique rather than open and volume center < 60 RAPN/year are risk factors of PSM. Another hypothesis is that easier procedures may have been left to young surgeons, since most of the centers in the study are academic.

As for more complex tumors, surgeons performing RAPN may cut more deeply into healthy parenchyma to reduce the risk of PSM. Whatever the reason, this result is robust even in multivariable analysis, after the inclusion of tumor size, overall operative time, blood loss and histology.

Like Takagi et al*.*^28^, we found that the RP approach (n = 168) was not a risk factor for PSM. They specifically compared RP with TP, though in a smaller sample size (48 RP vs. 290 TP) and found no significant difference between the two groups for PSM. Arora et al.^29^ also compared 99 RP-RAPN with 394 TP-RAPN and found no difference between the arms for margin status. Both of these studies suffered from the same limitations: retrospective design with a small sample size, and the absence of precise data about the surgeon’s experience and surgical technique for the tumorectomy.

As expected, a longer WIT is also a risk factor for PSM. WIT is a proxy for surgical experience and falls steeply in the early part of the learning curve, as described by Larcher et al.^30^. A longer WIT also reflects the occurrence of surgical difficulties during the procedure.

The fibrous stromal tumoral reaction and the presence, size and density of the tumor fibrous capsule vary according to histological sub-type. The reduced nature of these elements in chrRCCs may explain the higher frequency of PSMs in this sub-type^31^.

Finally, we set out to determine the impact of margins on oncological outcomes in this large, prospective, multicenter cohort of patients. To this end, further analyses are ongoing and will be published in due course.

Our study has several limitations that should be acknowledged. First, we deliberately excluded advanced tumors (cT3b-4, cN + , cM +), benign or multiple tumors and metastatic disease in order to establish a homogenous model of RAPN. Secondly, the follow-up was relatively short. Other series had a longer follow-up, especially that of Yossepowitch et al*.* (40.8mo)^25^. However, according to part of the literature, the median time to recurrence after RAPN ranges from 13.1 to 20 months^19,26,32,33^ and median time between partial nephrectomy and detection of local bed tumor recurrence was 23 months^21^. Experience is also an important factor to include. The number of RAPN already performed at the time of the first procedure recorded in UroCCR was not available for all surgeons, neither was the involvement of trainees. It was therefore impossible to assess the impact of experience on PSM. However, Larcher et al*.*^30^ highlighted that although surgeon experience was associated with a shorter warm ischemia time (WIT) and a lower probability of Clavien–Dindo complications (CD) ≥ 2, but it was not associated with a lower rate of PSM. We believe this cohort shows routine practice in university centers, where juniors are operating patients under seniors’ supervision so that EXP is a shallow concept. Finally, like numerous studies about RAPN, we lacked data concerning the length of the margin, the tumorectomy technique and the presence of adherent perinephric fat (APF), but these two last factors do not seem to be predictive of PSM in the literature^34–36^. We also lacked data for the treatment of the recurrence, and this treatment may have generated a selection bias by changing the results for MFS and OS. However, Brassier et al*.*^37^ showed that 40% of patients treated for local recurrence after PN by percutaneous ablation or surgical resection experienced disease recurrence within a median follow-up of 23 mo, which also means that only 60% of patients presented controlled disease after retreatment.

## Conclusions

Based on this large, French multicenter, retrospective series, PSM rates remained low after RAPN and had no impact on short-term oncological prognosis. PSM was associated with a longer WIT, chrRCC and surprisingly with a lower RENAL score.
